# Comparing SF-36 Scores Collected Through Web-Based Questionnaire Self-completions and Telephone Interviews: An Ancillary Study of the SENTIPAT Multicenter Randomized Controlled Trial

**DOI:** 10.2196/29009

**Published:** 2022-03-10

**Authors:** Ayşe Açma, Fabrice Carrat, Gilles Hejblum

**Affiliations:** 1 Sorbonne Université INSERM Institut Pierre Louis d'Épidémiologie et de Santé Publique Paris France; 2 Sorbonne Université INSERM Institut Pierre Louis d’Épidémiologie et de Santé Publique, AP-HP, Hôpital Saint-Antoine, Unité de Santé Publique Paris France

**Keywords:** Bias, Epidemiologic, Effect Modifier, Epidemiologic, Forms as Topic, Interviews, Telephone, Internet, Patient Reported Outcome Measures, Patient Satisfaction, Quality of Life, Surveys and Questionnaires

## Abstract

**Background:**

The 36-Item Short Form Health Survey (SF-36) is a popular questionnaire for measuring the self-perception of quality of life in a given population of interest. Processing the answers of a participant comprises the calculation of 10 scores corresponding to 8 scales measuring several aspects of perceived health and 2 summary components (physical and mental). Surprisingly, no study has compared score values issued from a telephone interview versus those from an internet-based questionnaire self-completion.

**Objective:**

This study aims to compare the SF-36 score values issued from a telephone interview versus those from an internet-based questionnaire self-completion.

**Methods:**

Patients with an internet connection and returning home after hospital discharge were enrolled in the SENTIPAT multicenter randomized trial on the day of discharge. They were randomized to either self-completing a set of questionnaires using a dedicated website (internet group) or providing answers to the same questionnaires administered during a telephone interview (telephone group). This ancillary study of the trial compared SF-36 data related to the posthospitalization period in these 2 groups. To anticipate the potential unbalanced characteristics of the responders in the 2 groups, the impact of the mode of administration of the questionnaire on score differences was investigated using a matched sample of individuals originating from the internet and telephone groups (1:1 ratio), in which the matching procedure was based on a propensity score approach. SF-36 scores observed in the internet and telephone groups were compared using the Wilcoxon-Mann-Whitney test, and the score differences between the 2 groups were also examined according to Cohen effect size.

**Results:**

Overall, 29.2% (245/840) and 75% (630/840) of SF-36 questionnaires were completed in the internet and telephone groups, respectively (*P*<.001). Globally, the score differences between groups before matching were similar to those observed in the matched sample. Mean scores observed in the telephone group were all above the corresponding values observed in the internet group. After matching, score differences in 6 out of the 8 SF-36 scales were statistically significant, with a mean difference greater than 5 for 4 scales and an associated mild effect size ranging from 0.22 to 0.29, and with a mean difference near this threshold for 2 other scales (4.57 and 4.56) and a low corresponding effect size (0.18 and 0.16, respectively).

**Conclusions:**

The telephone mode of administration of SF-36 involved an interviewer effect, increasing SF-36 scores. Questionnaire self-completion via the internet should be preferred, and surveys combining various administration methods should be avoided.

**Trial Registration:**

ClinicalTrials.gov NCT01769261; https://www.clinicaltrials.gov/ct2/show/record/NCT01769261

## Introduction

The 36-Item Short Form Health Survey (SF-36) is a popular questionnaire for measuring the self-perception of quality of life (QoL) in a given population of interest [1-3]: a query exploring the presence of the term *SF-36* in the title or the abstract of PubMed records retrieved 22,184 documents on September 28, 2021. SF-36 has been made available in 50 different languages, including French [4]. Although the SF-36 was initially developed as a paper-pencil format auto-questionnaire, the use of telephone interviews has also been reported for collecting SF-36 data [5-8]. Self-completion via the internet has been reported as a validated administration mode by Bell and Kahn [9] in 1996, and since then, with the spread of the internet and computers, several other computerized or internet-based formats have been applied in different studies [10-12].

Several randomized trials compared the SF-36 scores issued from different administration modes, such as paper versus the internet [13-17] or telephone versus paper [18-26]. Telephone interview is a common mode of questionnaire administration for several reasons, including the potential to increase response rate [24-26], practical convenience if other data of the study are already being collected via telephone, and exploring QoL in some special populations such as older patients. On the other hand, self-completion via the internet has advantages such as avoiding any potential response bias related to the interviewer effect [18], being potentially a simpler organization for collecting SF-36 data, and being associated with lower costs. However, and surprisingly, to our knowledge, no study has compared telephone interview and internet-based auto-questionnaire methods for collecting SF-36 data to investigate whether they can be used as alternative methods in mixed mode data collection procedures according to participant preferences and minimize the possible selection bias. This study investigated such questions in detail, owing to the availability of SF-36 data that had been collected in a multicenter randomized trial.

The SENTIPAT trial [27] explored the concept of sentinel patients who would voluntarily report their health evolution on a dedicated website. Participants enrolled in this trial were randomized to either the internet or the telephone group; patients in the internet group were invited to self-complete questionnaires on their health evolution after their hospital discharge via a dedicated website, whereas patients in the telephone group were invited to complete the same questionnaires through telephone interviews. However, 2 previous studies issued from the SENTIPAT trial have been reported: the first introduced an original questionnaire developed in the SENTIPAT study to investigate the opinion of patients about the organization of their hospital discharge [28], and the second introduced the I-Satis questionnaire, a questionnaire that was distributed in hospitals at a national level in France to investigate patient satisfaction at the time of the SENTIPAT trial [29]. As the SF-36 questionnaire was also included in the SENTIPAT trial, the corresponding collected data were a perfect opportunity to precisely investigate the influence of the mode of administration of the questionnaire on SF-36 scores. This investigation is the aim of the ancillary study of the SENTIPAT trial reported here.

## Methods

This research was an ancillary study of the multicenter, randomized SENTIPAT trial [27]. We took advantage of the trial to investigate the impact of the mode of administration of the SF-36 questionnaire on SF-36 scores.

### Population

Briefly, as previously reported [28,29], participants recruited consecutively from 5 different volunteer units (hepatogastroenterology, gastrointestinal enterology and nutrition, general and digestive surgery, infectious and tropical diseases, and internal medicine) of the Hôpital Saint-Antoine were enrolled in the SENTIPAT trial. Patients with internet access at home, aged ≥18 years, not cognitively impaired and without a behavioral disorder, speaking French, returning home after hospitalization, and not opposed to participating in the trial were eligible for inclusion.

Inpatients were enrolled on the day of hospital discharge by a clinical research technician of the trial. At that time, the patients were informed about the study. Eligible patients not opposed to participating in the study were randomized into two parallel groups—internet or telephone follow-up (inherently resulting in an open-label trial)—in a ratio of 1:1. On the basis of centralized randomization that allocated the eligible patient either to the internet or to the telephone group through a website and using an underlying permutation block randomization stratified by hospital unit, a computer-generated list of permutations was established by a statistician independent of the study. At the time of patient inclusion, the technician also collected baseline variables (length of stay, sex, age, relationship status, level of education, activity, and type of insurance). The patient was then informed and discharged with documents explaining the corresponding questionnaire administration. A total of 1680 eligible patients (840 randomized in the internet group and telephone group each) were enrolled in the SENTIPAT trial between February 25, 2013, and September 8, 2014.

### Ethics Approval

The SENTIPAT study was approved by the Comité de Protection des Personnes Île de France IX (decision CPP-IDF IX 12-014; June 12, 2012), the Comité Consultatif sur le Traitement de l’Information en matière de Recherche dans le domaine de la Santé (decision 12.365; June 20, 2012), and the Commission Nationale de l’Informatique et des Libertés (decision DR-2012-582; December 12, 2012). According to the French law in force at the time of the study, the formal consent of participants was waived and replaced by the following: patients received full information on their participation in the study, and the nonopposition of each participant in the study was notified (including date of nonopposition declaration) in the SENTIPAT study register.

### Survey Administration

Patients in the internet group had access to the French version of the SF-36 questionnaire 40 days after discharge on a website dedicated to SENTIPAT. Oral and written instructions had been delivered to these patients for a personal connection to the SENTIPAT website, and they received 1 reminder email per week for 3 weeks in case of nonresponse. Patients in the telephone group were interviewed by telephone approximately 42 days after discharge, and the data were simultaneously entered into the system by the interviewer using a website interface identical to that used in the internet group. The appointments for the telephone interviews of the patients in the telephone group were scheduled at the moment of patient inclusion, and up to 3 calls were tried whenever the first call did not reach the patient.

### SF-36 Questionnaire and Score Calculations

The 8 scale scores and the 2 summary scores of SF-36 were calculated according to the Medical Outcomes Study SF-36 French scoring manual [30]. The main lines of the corresponding process can be summarized as follows. The SF-36 questionnaire is composed of 36 items. Completion of the SF-36 questionnaire consists in choosing one of the proposed precoded answers for each of the 36 items in the questionnaire. The analysis of 35 items (an item that relates to the evolution of perceived health is not involved in any score calculation) comprises a structured calculation of 10 scores corresponding to 8 scales measuring several aspects of perceived health and 2 summary components. The eight scales and the corresponding number of questionnaire items involved are as follows: physical functioning (10 items), role-physical (RP; corresponding to role limitations because of physical problems; 4 items), bodily pain (3 items), general health (5 items), vitality (4 items), social functioning (2 items), role-emotional (corresponding to role limitations because of emotional problems; 3 items), and mental health (5 items). The raw score of each scale was computed by the algebraic sum of the corresponding item values (the values assigned to each precoded answer were calibrated) and then normalized to a score value ranging from 0 (lowest possible) to 100 (highest possible). According to the recommendations, scale score calculations were performed only if at least half of the items involved were answered, and in such a case, missing item data were treated with a person-specific approach that uses the average score of the completed items on the same scale. Finally, the two remaining scores, the physical and mental component summary scores, were obtained by assigning predefined specific weights to each of the 8 scale scores.

### Statistical Analyses

Bivariate analyses were performed using Fisher exact test or chi-square test of independence for categorical variables and the Wilcoxon-Mann-Whitney test for quantitative variables. The latter test was notably used to compare the SF-36 score differences between the internet and telephone groups. Several authors have discussed the task of interpreting observed differences in terms of *clinically meaningful* differences [31-33]. In this study, in addition to the abovementioned statistical test, the differences in SF-36 scores between the internet and telephone groups were also examined using two popular approaches: on the one hand, the effect size of the difference was considered according to Cohen's effect size index with corresponding small, medium, and large values at 0.2, 0.5, and 0.8, respectively [34]; on the other hand, we considered a threshold difference of 5 points, as proposed by Ware et al [33] for defining a clinically and socially relevant difference between 2 compared scores. The internal reliability of the SF-36 was evaluated by Cronbach's α coefficient calculation for the 8 scales and was considered acceptable if the α value was >.7. All statistical analyses of the study were performed using R freeware (version 3.3.3; R Foundation for Statistical Computing).

The difference between the observed SF-36 score estimates in responders of the internet group and responders of the telephone group may be mainly due to two features: (1) the difference in the mode of administration of the questionnaire, strictly speaking (self-completion of the patient via the internet vs completion of a research technician via a telephone interview with the patient), and (2) unbalanced characteristics of the individuals in the 2 groups issued from a selection bias of the responders (an unavoidable situation inherent to the modes of administration of the questionnaire). Assessing the respective impact of these 2 features on the observed differences between the SF-36 scores observed in internet and telephone responders is of primary importance, and to get more insight into this issue, we developed a procedure in which responders of the internet group were matched to similar responders of the telephone group according to their baseline characteristics, and we further examined how the score differences between the 2 groups changed in this matched sample, as compared with the score differences observed in the initial unmatched populations.

Internet responders were matched to telephone responders according to a propensity score–based procedure, and the R package MatchIt [35] was used to match each internet responder to the nearest telephone responder in a 1:1 ratio. The following baseline variables were included in the logistic regression model of the propensity score (propensity for being an internet responder vs being a telephone responder) as independent variables: age, length of stay, education, employment (unemployed because of health, retired or unemployed, job seeker, employed, and student), income, relationship status, and type of health insurance. We also forced each pair to be strictly identical according to three additional qualitative variables also included in the logistic regression model as independent variables: sex (male or female), type of hospitalization (conventional, weekly, or day-care hospitalization), and hospital ward (general and digestive surgery, gastroenterology and nutrition, hepatogastroenterology, infectious and tropical diseases, or internal medicine).

## Results

### Enrollment of the Participants in the SENTIPAT Study

**Figure 1:**
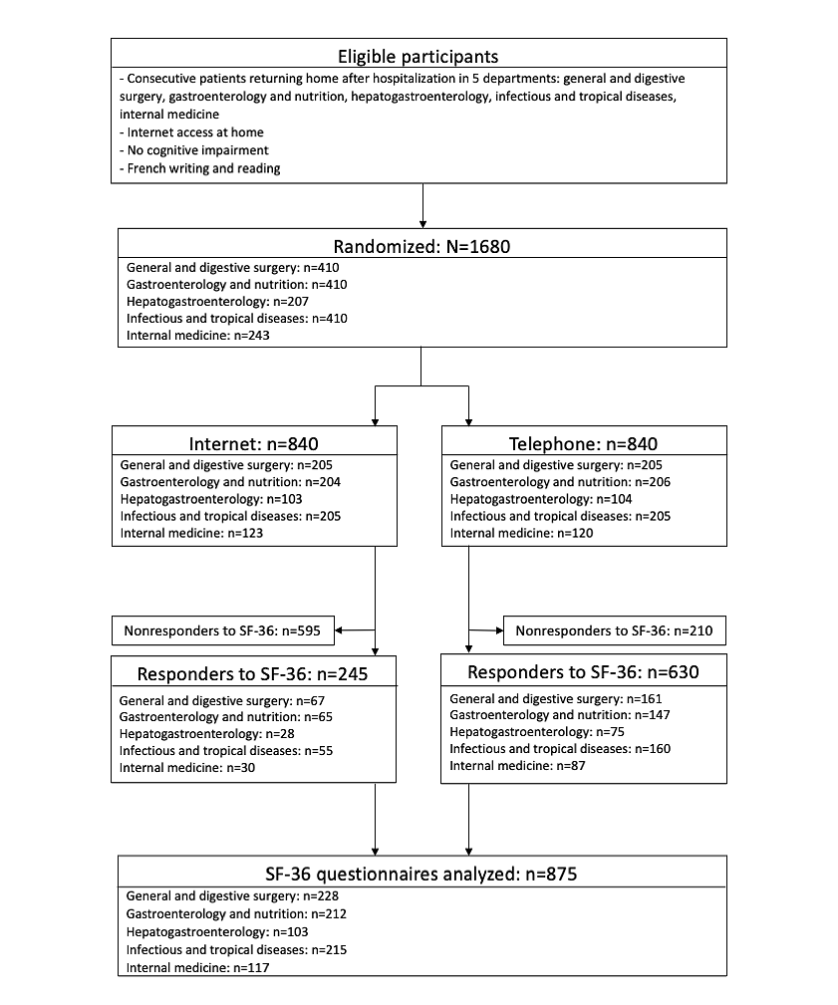
Flow of participants through the study. SF-36: 36-Item Short Form Health Survey.

Figure 1 presents the flowchart of the study. The randomization of the participants in either the internet or telephone group yielded the enrollment of 840 participants to the SENTIPAT study in each arm. Table 1 shows the baseline characteristics of the patients who constituted the population investigated in this study in each group and according to the responder or nonresponder status of the participants.

**Table 1:** Demographic characteristics of responders and nonresponders in the internet and telephone groups (N=1640).

Feature		Internet			Telephone	
		Responders (n=245)	Nonresponders (n=595)	Responders (n=630)		Nonresponders (n=210)
**Sex, n (%)**						
	Female	109 (44.5)	269 (45.2)	254 (40.3)		103 (49)
	Male	136 (55.5)	326 (54.8)	376 (59.7)		107 (51)
**Age (years)**						
	Values, mean	49.5	46.6	47.2		43.8
	Values, median (IQR)	50 (37-61)	47 (33-59)	47 (34-58)		41 (30-54)
**Length of stay (days)**						
	Values, mean	4.0	4.0	4.0		4.1
	Values, median (IQR)	1 (1-5)	1 (1-5)	1 (1-5)		1 (1-6)
**Type of hospitalization, n (%)**						
	Conventional	102 (41.6)	256 (43)	269 (42.7)		91 (43.3)
	1-day stay	120 (49)	285 (47.9)	297 (47.1)		103 (49.1)
	Week stay	23 (9.4)	54 (9.1)	64 (10.2)		16 (7.6)
**Ward, n (%)**						
	General and digestive surgery	67 (27.3)	138 (23.2)	161 (25.6)		44 (21)
	Gastroenterology and nutrition	65 (26.5)	139 (23.4)	147 (23.3)		59 (28.1)
	Hepatogastroenterology	28 (11.4)	75 (12.6)	75 (11.9)		29 (13.8)
	Infectious and tropical diseases	55 (22.4)	150 (25.2)	160 (25.4)		45 (21.4)
	Internal medicine	30 (12.2)	93 (15.6)	87 (13.8)		33 (15.7)
**Employment, n (%)**						
	Currently employed	158 (65)	353 (59.3)	375 (59.5)		132 (63.2)
	Job seeker	17 (7)	43 (7.2)	47 (7.5)		15 (7.2)
	Retired	47 (19.3)	98 (16.5)	101 (16)		29 (13.9)
	Student	6 (2.5)	38 (6.4)	48 (7.6)		17 (8.1)
	Does not work because of health	11 (4.5)	48 (8.1)	49 (7.8)		11 (5.3)
	Without work	2 (0.8)	9 (1.5)	8 (1.3)		4 (1.9)
	Other	2 (0.8)	6 (1)	2 (0.3)		1 (0.5)
**Type of employment, n (%)**						
	Farmer	0 (0)	1 (0)	0 (0)		0 (0)
	Self-employed or trader	4 (1.6)	25 (4.2)	27 (4.3)		11 (5.3)
	Manager	80 (32.7)	135 (22.7)	159 (25.2)		49 (23.4)
	Intermediate profession	39 (15.9)	91 (15.3)	105 (16.7)		31 (14.8)
	Middle-class occupation	52 (21.2)	135 (22.7)	123 (19.5)		55 (26.3)
	Employee	5 (2)	20 (3.4)	25 (4)		8 (3.8)
	Worker	42 (17.1)	83 (13.9)	92 (14.6)		22 (10.5)
	No work	23 (9.4)	105 (17.6)	99 (15.7)		33 (15.8)
**Level of education, n (%)**						
	Primary or less	18 (7.3)	58 (9.7)	47 (7.5)		31 (14.8)
	High school	75 (30.6)	193 (32.4)	178 (28.3)		60 (28.7)
	Superior short time	37 (15.1)	95 (16)	94 (14.9)		33 (15.8)
	Graduate or postgraduate	115 (46.9)	249 (41.8)	311 (49.4)		85 (40.7)
**Relationship status, n (%)**						
	Living alone^a^	103 (42)	291 (48.9)	293 (46.5)		121 (57.9)
	Living as a couple^b^	142 (58)	304 (51.1)	337 (53.5)		88 (42.1)
**Income level (€),^c^ n (%)**						
	<450	6 (2.4)	28 (4.7)	31 (4.9)		10 (4.8)
	450-1000	3 (1.2)	37 (6.2)	31 (4.9)		11 (5.3)
	1000-1500	17 (6.9)	61 (10.3)	51 (8.1)		17 (8.1)
	1500-2100	34 (13.9)	75 (12.6)	78 (12.4)		27 (12.9)
	2100-2800	26 (10.6)	70 (11.8)	66 (10.5)		25 (12)
	2800-4200	44 (18)	79 (13.3)	108 (17.1)		28 (13.4)
	≥4200	43 (17.6)	64 (10.8)	82 (13)		16 (7.7)
	No response	72 (29.4)	181 (30.4)	183 (29)		75 (35.9)
**Type of insurance, n (%)**						
	State medical help or universal health insurance	2 (0.8)	26 (4.4)	24 (3.8)		8 (3.8)
	Compulsory health insurance	15 (6.1)	43 (7.2)	43 (6.8)		26 (12.4)
	Compulsory health insurance plus complementary private health insurance	228 (93.1)	526 (88.4)	563 (89.4)		175 (83.7)

^a^Single, widowed, divorced, or separated.

^b^Married, living together under a civil solidarity pact, or simply living together without legal ties.

^c^€1 (in 2013)=US $0.71 (in 2022).

### Response Rate, Delay of Questionnaire Completion, and Internal Validity of Questionnaire Completion

The response rate observed in the intervention group (245/840, 29.2%) was significantly lower (*P*<.001) than that observed in the telephone group (630/840, 75%). The median (IQR) delay between hospital discharge and questionnaire completion was 42 (40-46) and 42 (42-46) days in responders of the internet and telephone groups, respectively.

In terms of internal validity of questionnaire completion, Cronbach's α values calculated for each of the 8 scales comprising the SF-36 form in the internet and telephone groups (Multimedia Appendix 1) were all >.7, which is the threshold value considered as acceptable.

### Assessment of the Procedure Matching the Responders of the Internet Group With Responders of the Telephone Group

**Figure 2:**
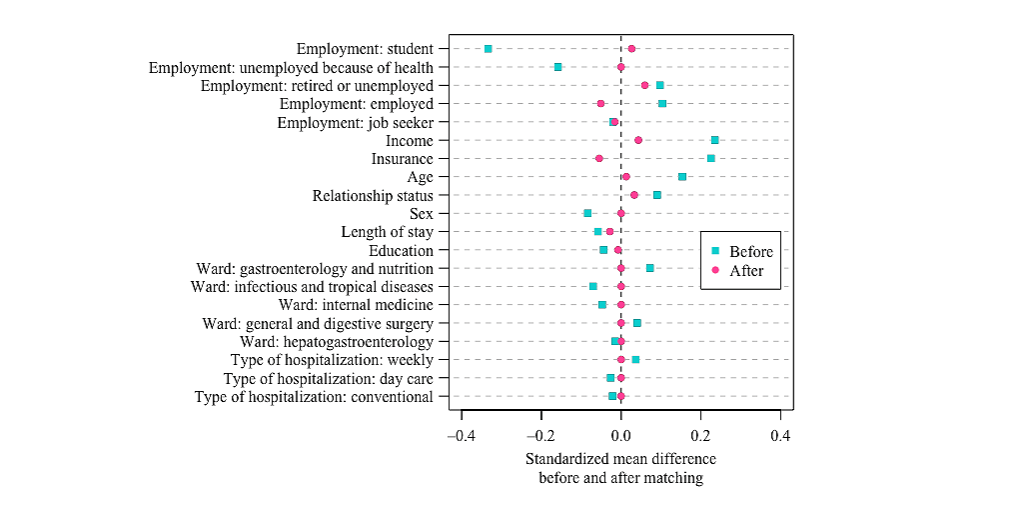
Differences in baseline variables between the internet and telephone responders before and after the matching procedure.

The matching procedure matched the 245 responders in the internet group (no individual was dropped) with 245 individuals in the telephone group. The standardized mean difference of the global distance between internet and telephone groups was 0.4167 and 0.0215 before and after matching, respectively, with a corresponding balance improvement of 95%. Figure 2 details the standardized mean differences between the internet and telephone groups observed on baseline variables before and after the matching procedure. The differences between the internet and telephone groups before matching were globally dramatically decreased after matching, indicating that the matching procedure successfully yielded two populations, internet and telephone, which were highly comparable in terms of the baseline variables.

### SF-36 Score Differences According to the Mode of Administration of the Questionnaire

**Figure 3:**
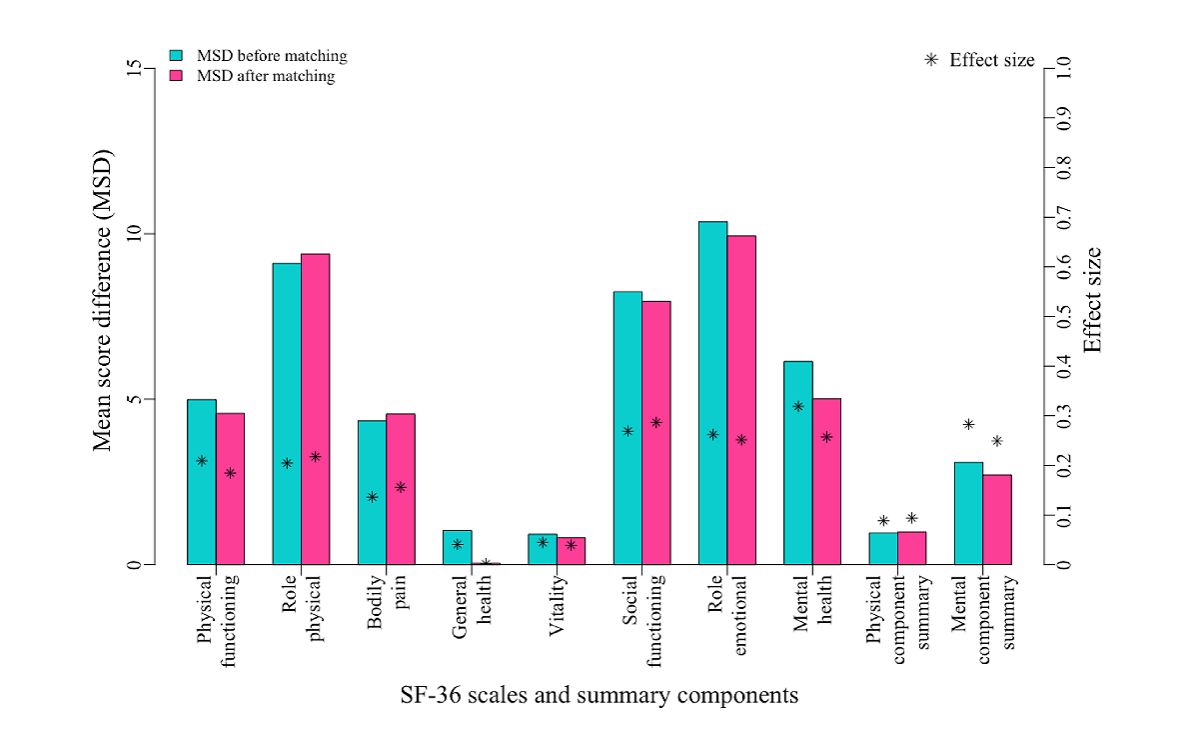
Observed mean score differences (telephone–internet) of SF-36 scales and summary components before and after matching. SF-36: 36-Item Short Form Health Survey.

Figure 3 shows the differences between the internet and telephone groups, before and after matching, for the 8 scales and the 2 summary measures composing SF-36. Figure 3 indicates that the matching procedure had a limited impact on the differences observed between the internet and telephone groups in each of the components of SF-36; regardless of the value of the difference before matching, the corresponding difference after matching appeared similar. Importantly, the means observed in the telephone group were all above the corresponding values observed in the internet group.

Table 2 details the results observed after matching. The mean difference between the internet and telephone groups was >5 (threshold recommended for declaring that the difference corresponds to a significant clinical status) for four scales (RP, social functioning, role-emotional, and mental health) with an associated effect size ranging from 0.22 to 0.29, close to the value of 0.2, which is defined as a small effect size by Cohen. Moreover, the difference approached this threshold for 2 other scales (4.57 and 4.56 for physical functioning and bodily pain, respectively), with small corresponding effect size, 0.18 and 0.16, respectively. The abovementioned 6 differences were all statistically significant (Table 2). In contrast, the observed mean difference between telephone and internet was low for the remaining 2 scales (0.04 and 0.82 for general health and vitality, respectively) and not significant. When examining the physical and the mental component summary, the difference was 0.99 and 2.72, respectively, the latter difference being statistically significant and with an associated effect size at 0.25.

**Table 2:** The 36-Item Short Form Health Survey scores in the internet and telephone groups after matching (N=245 each).

Scale or component summary and group		Score, median (IQR)	Score, mean (SD)	Score, mean (95% CI)	Score difference (telephone−internet)					
					*P* value		Mean difference		Effect size	
**Physical functioning**						.02		4.57		0.18
	Internet	85 (65-95)	76.08 (24.56)	76.08 (72.92-79.08)						
	Telephone	90 (70-100)	80.65 (24.93)	80.65 (77.47-83.71)						
**Role-physical**						.002		9.39		0.22
	Internet	50 (0-100)	51.53 (41.67)	51.53 (46.22-56.73)						
	Telephone	100 (0-100)	60.92 (44.59)	60.92 (55.31-66.43)						
**Bodily pain**						.045		4.56		0.16
	Internet	72 (41-100)	66.84 (26.12)	66.84 (63.55-70.11)						
	Telephone	84 (41-100)	71.40 (32.23)	71.40 (67.42-75.40)						
**General health**						.99		0.04		0.00
	Internet	57 (42-72)	55.10 (20.47)	55.10 (52.57-57.65)						
	Telephone	57 (37-77)	55.15 (25.90)	55.15 (51.96-58.34)						
**Vitality**						.57		0.82		0.04
	Internet	50 (35-65)	48.29 (20.16)	48.29 (45.78-50.80)						
	Telephone	50 (35-65)	49.10 (21.30)	49.10 (46.41-51.78)						
**Social functioning**						<.001		7.96		0.29
	Internet	75 (50-100)	71.17 (24.27)	71.17 (68.16-74.18)						
	Telephone	100 (62.5-100)	79.13 (31.24)	79.13 (75.15-82.96)						
**Role-emotional**						.002		9.93		0.25
	Internet	100 (33.33-100)	67.89 (39.04)	67.89 (63.13-72.65)						
	Telephone	100 (66.66-100)	77.82 (39.84)	77.82 (72.79-82.59)						
**Mental health**						.002		5.01		0.26
	Internet	64 (52-80)	63.56 (18.77)	63.56 (61.21-65.91)						
	Telephone	72 (56-84)	68.57 (20.20)	68.57 (65.94-71.10)						
**Physical component summary**						.18		0.99		0.09
	Internet	44.95 (37.27-53.30)	44.48 (10.04)	44.48 (43.20-45.75)						
	Telephone	48.33 (37.81-54.43)	45.47 (11.05)	45.47 (44.09-46.82)						
**Mental component summary**						.02		2.72		0.25
	Internet	47.49 (35.37-52.60)	44.68 (10.62)	44.68 (43.34-46.01)						
	Telephone	50.86 (41.81-55.50)	47.40 (11.15)	47.40 (46.01-48.76)						

## Discussion

### The Opportunity to Investigate the Influence of the Mode of Administration of the Questionnaire on SF-36 Scores

To our knowledge, this study is the first reported to date to compare SF-36 questionnaire data collected either via telephone interviews or via self-completion on a dedicated internet website. More precisely, the availability of SF-36 data collected in the SENTIPAT trial provided a perfect opportunity to precisely investigate the influence of the mode of administration of the questionnaire on SF-36 scores. This investigation was the aim of the ancillary study of the SENTIPAT trial reported here and constitutes the major contribution of our report. This investigation has benefited from 3 main strengths. First, the study is based on a randomized trial with a substantial number of patients included in both arms. Second, the population under study had a large patient case mix variability because of the fact that patients originated from 5 very different hospital wards. The third strength of the study is the construction of a matched subsample of comparable responders in the 2 arms according to baseline variables to mitigate the impact of an unavoidable selection bias of responders as much as possible.

### Principal Findings

Figure 2 shows that the matching procedure highly succeeded in composing a sample of similar match-paired patients; however, the very modest impact of this matching procedure on modifying the initial score differences between the scores in the internet and telephone groups (Figure 3) highly suggests that the score differences between internet and telephone groups are mainly attributable to the mode of administration, strictly speaking, with a minor impact of selection bias issues. In addition, and importantly, the scores in the telephone group were always higher than those in the internet group (Figure 3; Table 2), likely reflecting another type of bias associated with the telephone interview mode of administration: the interviewer effect.

For all but 2 out of 8 scales, the mean difference in scores between the groups was statistically significant and >4.5 points (Table 2), and several comments have to be made about this statement. It is worth recalling that misinterpretations of *P* values are very common [36,37]. A statistically significant score difference was not systematically considered as meaningful by the authors [38,39], and Ware et al [33] had initially proposed a 5-point difference between 2 SF-36 scores as a threshold value for a clinically and socially relevant difference. In our view, considering effect size is an appropriate approach for examining the relevance of score differences as such a perspective takes into account the variability of the measures and not only a rough mean difference threshold. Interestingly, as shown in Table 2, even if there were substantial mean score differences between the 2 different modes of administration in most of the scales, these differences were all related to a small effect size in 8 scales and in 2 component summaries of SF-36 according to the effect size index classification proposed by Cohen [34]. Cohen defined the small effect size as “noticeably smaller than medium which represents an effect visible to naked eye of a careful observer but also not so small as to be trivial.” On the one hand, the effect size perspective considerably softens the observed differences between the internet and telephone groups and raises concerns about the relevance of considering a mean difference of 5 points as the main critical element of comparison between 2 scores. Moreover, our analyses also indicate that in studies involving a substantially variable population, only very large mean differences in scores would be considered meaningful when adopting the effect size perspective, highly limiting the usefulness of SF-36 in such studies. On the other hand, some mean differences in scores observed in our study and most likely attributable to the interviewer effect are not negligible. For example, in patients with chronic hepatitis C, Younossi et al [40] reported a mean value of the RP scale at 74.4 and 79.6 in patients with advanced and none to mild fibrosis, respectively (*P*=.002). Therefore, the differences for the RP scale, likely attributable to the SF-36 mode of administration observed in this study (51.5 and 60.9 in the internet and telephone groups, respectively; *P*=.002; Table 2), are at least comparable with those attributable to substantially different health states reported in other studies.

### Limitations

The main limitation of the study concerns the selection bias related to responder status in both arms; however, such a bias is inherent to the 2 corresponding modes of administration, and this bias is likely different from one mode of administration to the other. In this study, selection biases were mitigated as much as possible by conducting a part of the analyses in a matched subpopulation of responders. A detailed analysis comparing the scores observed in the whole set of responders (before matching) and in a subpopulation enhancing the similarity of the compared individuals (after matching) constitutes an important strength of the study. Our results evidence an interviewer effect, which artificially increased SF-36 scores when the questionnaire was administered through a telephone interview. Therefore, the telephone interview as a mode of administration of SF-36 cumulates two types of bias: the unavoidable associated selection bias of responders and the interviewer effect, which is discussed in more detail in the following sections. In general, several methods can be used for mitigating the selection bias of responders as much as possible: one takes advantage of the distribution of baseline values observed in the responders and nonresponders to correct initial responder estimates to estimates more representative of the whole population under study [41]. In contrast, the interviewer effect raises many more concerns as the corresponding bias cannot be removed.

For the rest, some of the estimates reported here raise concerns in terms of generalizability and should only be viewed as minor side results that were required in the global process of the main goal of the study, which was to investigate the impact of the mode of administration of the SF-36 questionnaire on the collected scores. For example, the response rates reported here should not be considered emblematic of the corresponding modes of administration of the questionnaires. As detailed below in the *Response Rates According to the Mode of Administration* subsection, response rates reported in any study, including ours, are hardly generalizable as such rates likely depend on many characteristics of the survey design. Similarly, the reader should keep in mind that the SF-36 scores collected here reflect the QoL of a particular population of patients admitted in 5 departments of a French university hospital, and these scores are not generalizable to other populations.

### Comparison With Prior Works

#### Interviewer Effect

To our knowledge, this study is the only one to date that compared modes of administration of SF-36 on a matched sample of responders to mitigate—as much as possible—the inherent lack of initial comparability of responders according to the mode of administration of the questionnaire. Nevertheless, our results are in agreement with previous studies that reported higher SF-36 scores when administered by telephone than those issued from a mailed paper mode of administration [18,21,22,24-26]. Similarly, Lyons et al [42,43] reported higher scores issued from a face-to-face interview administration than those issued from a mailed paper self-completion of the SF-36 questionnaire. Altogether, our results and those of previous studies suggest that as compared with patients’ self-completion, the introduction of an interviewer likely acts as a veil that somehow embellishes patients’ QoL-reported perception. Internet self-completion avoids any potential bias of responses related to an interviewer effect [44], and patients are more likely to freely express their opinions [45] on websites covering anonymity than through telephone. Therefore, self-completion (internet or paper) should be preferred for collecting SF-36 data, as the involvement of a third party appears to artificially increase the scores. In any case, our study indicates that an accurate comparison of different scores requires at least avoiding modes of administration of SF-36 mixing self-completion and interview.

#### Response Rates According to the Mode of Administration

Despite the reminders sent to the patients, the internet group response rate (245/840, 29.2%) to the survey was dramatically lower than that of the telephone group (630/840, 75%). Blumenberg and Barros [46] explored the response rate differences between web and alternative data collection methods for public health research; considering the 9 papers comparing web self-completion with telephone and with a sample size >100, which were selected in their review, the median and range of the response rates reported for web and telephone were 23% and 2% to 68% and 40% and 8% to 71%, respectively. Similarly, a recent meta-analysis comparing response rates of web surveys with those obtained with other modes of administration [47] indicated that the results were stable when compared with a similar analysis conducted 10 years earlier: web surveys still yielded lower response rates than other modes, with a mean difference of 12% and large heterogeneity in the differences observed. No study compared telephone and internet administration modes for SF-36; however, the participation rates reported in studies that compared several modes of administration of SF-36 substantially varied from one study to another. For example, the response rate with the telephone was significantly higher than that with postal mail in the study by Wettergren et al [26] (77% and 63%, respectively; *P*<.001), as well as in the study by Perkins and Sanson-Fisher [24] (85% and 68%, respectively; *P*<.001), whereas corresponding response rates were similar in the study by McHorney et al [23] (65.3% and 65.1%, respectively; *P*=.68) and in the study by Bursik et al [18] (71% and 68%, respectively; *P*=.48). In addition, the participation rate observed in our study in the internet group was close to that of Basnov et al [13], who reported a lower response rate in the internet group than that observed in the paper group (23% vs 76%, respectively).

In our view, the numeric value of the difference between the response rates observed in the 2 modes of administration of the present survey should be considered as a minor side result. Indeed, the heterogeneity of the comparisons reported in reviews [46,47] mostly reflects the fact that the differences between response rates collected via the internet versus other methods of administration reported in any survey are difficult to interpret and are not generalizable at all: the modes of administration include underlying elements of the whole survey process for which the impact on participation rate is hardly assessable or even describable, such as the internet website design in terms of its attractiveness or convenience or the detailed procedure for reaching participants by telephone. For example, the relatively high rate of participation in the telephone group observed in this study is likely related to the fact that the schedule of the telephone interview was arranged with each participant at the moment of his or her enrollment, and moreover, up to 3 calls were tried whenever the participant was not reached at the first phone call. In addition, many other features, such as the age distribution of the target population of the survey, might influence the observed response rates according to the mode or modes of administration of the questionnaire. In the end, internet use and use of telephones have evolved considerably since the completion of the SENTIPAT study. Such changes include not only technological aspects but also the growing importance of the abovementioned uses for many purposes throughout society. In particular, the COVID-19 crisis had a substantial impact on such matters [48,49]. Therefore, these changes are an additional element for limiting the interest in the intrinsic value of response rates, and it would be hazardous to consider that the participation rates in any web and telephone survey made before the COVID-19 period would be replicable whenever a similar survey would be conducted nowadays.

### Conclusions

As compared with the mode of administration based on telephone interviews, the response rate of volunteer patients communicating their SF-36 data via the internet was much lower; however, our study indicates that a substantial proportion of hospitalized patients volunteered to actively document their health data via the internet. Most of all, the study indicates that the telephone interviewer might be viewed as an intermediate subjective pattern in the collection of patient data, resulting in a nonnegligible increase in SF-36 scores. Therefore, self-administration of SF-36 should be preferred, including via the internet, which is likely a low-cost method. Importantly, the results of this study also strongly advocate avoiding the conduction of surveys combining methods of SF-36 administration that mix self-reporting and interviews.

## Appendix

Multimedia Appendix 1 Internal reliability of the 36-Item Short Form Health Survey in the internet and telephone groups.

Multimedia Appendix 2 CONSORT-eHEALTH checklist (V 1.6.1).

## Abbreviations

QoL: quality of life
RP: role-physical
SF-36: 36-Item Short Form Health Survey
