# Supplementary material for: Comparing SF-36 Scores Collected Through Web-Based Questionnaire Self-completions and Telephone Interviews: An Ancillary Study of the SENTIPAT Multicenter Randomized Controlled Trial
Source: J Med Internet Res. 2022 Mar 10;24(3):e29009. doi: 10.2196/29009 (PMC8949688; doi:10.2196/29009)
Supplement: Multimedia Appendix 1 [file jmir_v24i3e29009_app1.pdf]

Multimedia Appendix 1 to:

Comparing SF-36 Scores Collected Through a Web-Based Questionnaire Self-completion and a Telephone Interview: an Ancillary Study of the SENTIPAT Multicenter Randomized Controlled Trial

Ayşe Açma[1], Fabrice Carrat[2], and Gilles Hejblum[1§‡]

[1]Sorbonne Université, INSERM, Institut Pierre Louis d'Épidémiologie et de Santé Publique, F75012, Paris, France

[2]Sorbonne Université, INSERM, Institut Pierre Louis d'Épidémiologie et de Santé Publique, AP-HP, Hôpital Saint-Antoine, Unité de Santé Publique, F75012, Paris, France

[§]Corresponding author: gilles.hejblum@inserm.fr

[‡]on behalf of the SENTIPAT study group

Additional Table A1: Internal reliability of SF-36 in the Internet and Telephone group.

SF-36 Scale	Cronbach's alpha (95% confidence interval)	
	Internet group	Telephone group
PF, physical functioning	0.919 (0.903–0.933)	0.898 (0.885–0.909)
RP, role physical	0.861 (0.830–0.887)	0.931 (0.922–0.939)
BP, bodily pain	0.906 (0.879–0.927)	0.933 (0.921–0.943)
GH, general health	0.797 (0.754–0.835)	0.717 (0.681–0.751)
VT, vitality	0.852 (0.819–0.880)	0.714 (0.677–0.750)
SF, social functioning	0.879 (0.845–0.906)	0.943 (0.933–0.951)
RE, role emotional	0.791 (0.741–0.832)	0.954 (0.947–0.960)
MH, mental health	0.916 (0.898–0.932)	0.888 (0.874–0.901)
